# Supplementary material for: First Proteomic Approach to Identify Cell Death Biomarkers in Wine Yeasts during Sparkling Wine Production
Source: Microorganisms. 2019 Nov 8;7(11):542. doi: 10.3390/microorganisms7110542 (PMC6920952; doi:10.3390/microorganisms7110542)
Supplement: Supplementary file 1 [file microorganisms-07-00542-s001.zip › Table S1.pdf]

Table S1. Average values of score and peptides detected in apoptosis and autolysis proteins in *S. cerevisiae* P29 and G1. Values identified in each study condition (PC: pressure condition and NPC: non-pressure condition) and each sampling time (T1: middle of the second fermentation and T2: one month after it). Score values were calculated by summing the Xcorr values of each peptide detected. n.f.; not found.

APOPTOSIS

**Condition**	**PCT1**				**PCT2**				**NPCT1**				**NPCT2**			
**Strain**	***P29***		***G1***		***P29***		***G1***		***P29***		***G1***		***P29***		***G1***	
**Protein**	***Score***	***Peptides***	***Score***	***Peptides***	***Score***	***Peptides***	***Score***	***Peptides***	***Score***	***Peptides***	***Score***	***Peptides***	***Score***	***Peptides***	***Score***	***Peptides***
Bir1p	n.f.	n.f.	n.f.	n.f.	n.f.	n.f.	n.f.	n.f.	n.f.	n.f.	2.49	3	n.f.	n.f.	n.f.	n.f.
Cpr3p	16.64	4	116.93	9	13.18	3	n.f.	n.f.	73.96	7	99.71	8	40.82	7	7.51	2
Esp1p	n.f.	n.f.	3.20	2	n.f.	n.f.	n.f.	n.f.	5.19	4	n.f.	n.f.	n.f.	n.f.	n.f.	n.f.
Fis1p	n.f.	n.f.	n.f.	n.f.	n.f.	n.f.	n.f.	n.f.	6.44	2	n.f.	n.f.	10.67	4	n.f.	n.f.
Mca1p	n.f.	n.f.	n.f.	n.f.	17.16	3	n.f.	n.f.	14.00	4	8.09	4	3.63	4	n.f.	n.f.
Nma111p	n.f.	n.f.	4.60	4	n.f.	n.f.	n.f.	n.f.	8.58	5	n.f.	n.f.	n.f.	n.f.	n.f.	n.f.
Oye2p	44.57	19	191.70	23	107.6	21	n.f.	n.f.	216.09	22	65.17	19	10.85	8	n.f.	n.f.
Oye3p	n.f.	n.f.	n.f.	n.f.	n.f.	n.f.	n.f.	n.f.	27.08	7	8.17	2	5.98	5	28.51	9
Por1p	n.f.	n.f.	n.f.	n.f.	122.6	14	n.f.	n.f.	34.41	8	8.78	2	62.13	10	6.49	2
Rny1p	n.f.	n.f.	n.f.	n.f.	18.58	6	n.f.	n.f.	6.84	3	9.82	2	18.65	5	n.f.	n.f.
Tdh2p	302.38	31	627.55	34	527.6	32	335.89	24	663.28	37	688.35	38	316.2	38	261.56	21
Tdh3p	282.63	33	706.64	38	549.2	35	336.58	24	635.62	36	699.78	39	283.51	38	263.41	22
Ysp2p	n.f.	n.f.	n.f.	n.f.	n.f.	n.f.	n.f.	n.f.	2.06	2	n.f.	n.f.	n.f.	n.f.	n.f.	n.f.

AUTOLYSIS

**Condition**	**PCT1**				**PCT2**				**NPCT1**				**NPCT2**			
**Strain**	***P29***		***G1***		***P29***		***G1***		***P29***		***G1***		***P29***		***G1***	
**Protein**	***Score***	***Peptides***	***Score***	***Peptides***	***Score***	***Peptides***	***Score***	***Peptides***	***Score***	***Peptides***	***Score***	***Peptides***	***Score***	***Peptides***	***Score***	***Peptides***
Aap1p	4.02	2	20.40	5	n.f.	n.f.	n.f.	n.f.	49.47	25	52.32	19	n.f.	n.f.	3.21	2

Ai4p	n.f.	n.f.	n.f.	n.f.	n.f.	n.f.	n.f.	n.f.	2.33	3	2.09	2	n.f.	n.f.	n.f.	n.f.
Ai5_Alphap	n.f.	n.f.	n.f.	n.f.	n.f.	n.f.	n.f.	n.f.	n.f.	n.f.	2.03	2	n.f.	n.f.	n.f.	n.f.
Ams1p	n.f.	n.f.	n.f.	n.f.	12.52	4	n.f.	n.f.	107.36	24	n.f.	n.f.	n.f.	n.f.	n.f.	n.f.
Ape1p	16.94	10	48.42	13	44.87	10	n.f.	n.f.	68.70	19	31.53	11	30.21	13	17.40	6
Ape2p	2.33	2	53.22	15	n.f.	n.f.	n.f.	n.f.	80.10	28	33.12	11	n.f.	n.f.	22.38	7
Ape3p	15.39	7	154.71	17	157.8	13	n.f.	n.f.	73.88	15	91.06	23	44.97	18	15.37	5
Ape4p	n.f.	n.f.	n.f.	n.f.	n.f.	n.f.	n.f.	n.f.	10.93	4	9.11	3	n.f.	n.f.	n.f.	n.f.
Arx1p	n.f.	n.f.	n.f.	n.f.	n.f.	n.f.	n.f.	n.f.	40.73	11	n.f.	n.f.	n.f.	n.f.	n.f.	n.f.
Atg4p	n.f.	n.f.	n.f.	n.f.	n.f.	n.f.	n.f.	n.f.	22.16	6	14.50	3	n.f.	n.f.	n.f.	n.f.
Axl1p	n.f.	n.f.	n.f.	n.f.	n.f.	n.f.	n.f.	n.f.	3.17	3	2.29	2	n.f.	n.f.	n.f.	n.f.
Bgl2p	30.33	9	40.10	6	39.39	15	96.61	7	90.62	13	117.45	12	41.1	18	63.48	9
Cps1p	3.12	2	20.79	6	n.f.	n.f.	n.f.	n.f.	59.13	15	53.54	10	n.f.	n.f.	n.f.	n.f.
Cym1p	n.f.	n.f.	n.f.	n.f.	n.f.	n.f.	n.f.	n.f.	7.53	3	2.86	2	n.f.	n.f.	n.f.	n.f.
Dap2p	n.f.	n.f.	n.f.	n.f.	n.f.	n.f.	n.f.	n.f.	7.21	4	2.42	2	n.f.	n.f.	n.f.	n.f.
Dcw1p	n.f.	n.f.	n.f.	n.f.	n.f.	n.f.	n.f.	n.f.	15.51	3	11.99	2	n.f.	n.f.	n.f.	n.f.
Dfg5p	n.f.	n.f.	n.f.	n.f.	n.f.	n.f.	n.f.	n.f.	6.61	3	4.01	2	n.f.	n.f.	n.f.	n.f.
Dna2p	2.21	2	n.f.	n.f.	n.f.	n.f.	n.f.	n.f.	n.f.	n.f.	n.f.	n.f.	n.f.	n.f.	n.f.	n.f.
Dse4p	5.11	3	n.f.	n.f.	n.f.	n.f.	n.f.	n.f.	106.24	18	35.03	7	n.f.	n.f.	9.36	2
Dss1p	n.f.	n.f.	n.f.	n.f.	n.f.	n.f.	n.f.	n.f.	6.44	2	4.37	2	n.f.	n.f.	n.f.	n.f.
Dug1p	34.46	18	190.59	18	3.69	3	n.f.	n.f.	83.99	24	100.93	27	15	13	55.60	18
Dug2p	n.f.	n.f.	n.f.	n.f.	n.f.	n.f.	n.f.	n.f.	9.66	6	n.f.	n.f.	n.f.	n.f.	n.f.	n.f.
Ecm14p	n.f.	n.f.	n.f.	n.f.	n.f.	n.f.	n.f.	n.f.	4.47	2	n.f.	n.f.	n.f.	n.f.	n.f.	n.f.
Ecm38p	n.f.	n.f.	n.f.	n.f.	n.f.	n.f.	n.f.	n.f.	4.91	2	n.f.	n.f.	n.f.	n.f.	n.f.	n.f.
Esp1p	n.f.	n.f.	3.20	2	n.f.	n.f.	n.f.	n.f.	5.19	4	n.f.	n.f.	n.f.	n.f.	n.f.	n.f.
Exg1p	35.36	15	62.24	11	21.07	7	109.86	10	150.86	23	136.97	19	35.07	15	53.57	11
Exg2p	n.f.	n.f.	5.17	2	n.f.	n.f.	n.f.	n.f.	7.80	3	7.95	2	n.f.	n.f.	n.f.	n.f.
Fra1p	2.13	2	n.f.	n.f.	n.f.	n.f.	n.f.	n.f.	8.29	3	n.f.	n.f.	n.f.	n.f.	n.f.	n.f.
Lap2p	n.f.	n.f.	7.54	2	n.f.	n.f.	n.f.	n.f.	23.48	10	12.30	5	n.f.	n.f.	n.f.	n.f.

Lap3p	n.f.	n.f.	14.71	5	n.f.	n.f.	n.f.	n.f.	55.28	14	18.38	5	n.f.	n.f.	n.f.	n.f.
Map1p	10.02	5	n.f.	n.f.	n.f.	n.f.	n.f.	n.f.	90.10	16	130.70	22	n.f.	n.f.	n.f.	n.f.
Mas1p	n.f.	n.f.	28.04	5	n.f.	n.f.	n.f.	n.f.	3.52	3	12.98	6	n.f.	n.f.	n.f.	n.f.
Mas2p	n.f.	n.f.	7.38	4	n.f.	n.f.	n.f.	n.f.	8.97	4	8.69	3	n.f.	n.f.	8.02	3
Mca1p	n.f.	n.f.	n.f.	n.f.	17.16	3	n.f.	n.f.	14.00	4	8.09	4	3.63	4	n.f.	n.f.
Nma111p	n.f.	n.f.	4.60	4	n.f.	n.f.	n.f.	n.f.	8.58	5	n.f.	n.f.	n.f.	n.f.	n.f.	n.f.
Otu1p	n.f.	n.f.	n.f.	n.f.	n.f.	n.f.	n.f.	n.f.	19.44	4	4.94	2	n.f.	n.f.	n.f.	n.f.
Pep4p	74.02	16	96.80	14	25.46	12	112.56	12	152.69	18	228.73	21	28.19	16	52.27	12
Pim1p	n.f.	n.f.	n.f.	n.f.	n.f.	n.f.	n.f.	n.f.	37.21	11	13.15	4	n.f.	n.f.	n.f.	n.f.
Prb1p	27.06	8	26.64	8	64.6	8	8.37	2	78.04	12	46.67	11	21.6	10	4.65	2
Prc1p	13.79	6	68.39	9	n.f.	n.f.	45.54	9	96.61	15	87.29	11	n.f.	n.f.	35.98	10
Prd1p	11.13	6	16.48	5	n.f.	n.f.	n.f.	n.f.	75.51	24	15.75	6	n.f.	n.f.	n.f.	n.f.
Pre10p	n.f.	n.f.	10.05	5	n.f.	n.f.	n.f.	n.f.	29.01	5	n.f.	n.f.	n.f.	n.f.	n.f.	n.f.
Pre1p	n.f.	n.f.	n.f.	n.f.	n.f.	n.f.	n.f.	n.f.	18.60	5	8.40	3	n.f.	n.f.	n.f.	n.f.
Pre2p	n.f.	n.f.	n.f.	n.f.	n.f.	n.f.	n.f.	n.f.	20.51	7	n.f.	n.f.	n.f.	n.f.	n.f.	n.f.
Pre3p	9.11	5	36.58	6	8.8	3	n.f.	n.f.	27.66	9	18.59	8	n.f.	n.f.	6.66	2
Pre4p	n.f.	n.f.	7.66	2	n.f.	n.f.	n.f.	n.f.	12.47	5	3.75	2	n.f.	n.f.	n.f.	n.f.
Pre5p	7.02	4	15.77	3	n.f.	n.f.	n.f.	n.f.	47.73	13	13.26	4	n.f.	n.f.	n.f.	n.f.
Pre6p	n.f.	n.f.	23.96	3	15.06	3	n.f.	n.f.	71.41	11	26.74	5	n.f.	n.f.	n.f.	n.f.
Pre7p	n.f.	n.f.	n.f.	n.f.	21.12	6	n.f.	n.f.	22.51	6	5.81	3	n.f.	n.f.	n.f.	n.f.
Pre8p	5.01	4	9.20	3	10.52	5	n.f.	n.f.	31.27	10	26.42	9	4.22	4	n.f.	n.f.
Pre9p	16.67	8	31.21	7	6.2	3	n.f.	n.f.	40.88	13	38.38	12	3.07	2	n.f.	n.f.
Pup1p	n.f.	n.f.	n.f.	n.f.	n.f.	n.f.	n.f.	n.f.	18.24	4	20.63	3	n.f.	n.f.	n.f.	n.f.
Pup2p	30.98	12	24.13	7	n.f.	n.f.	n.f.	n.f.	77.71	16	122.73	15	2.13	3	n.f.	n.f.
Pup3p	8.34	3	9.58	2	n.f.	n.f.	n.f.	n.f.	33.25	8	21.06	5	n.f.	n.f.	n.f.	n.f.
Rad2p	n.f.	n.f.	n.f.	n.f.	n.f.	n.f.	n.f.	n.f.	2.09	2	n.f.	n.f.	n.f.	n.f.	n.f.	n.f.
Rai1p	n.f.	n.f.	n.f.	n.f.	n.f.	n.f.	n.f.	n.f.	2.53	2	n.f.	n.f.	n.f.	n.f.	n.f.	n.f.
Rex2p	n.f.	n.f.	n.f.	n.f.	n.f.	n.f.	n.f.	n.f.	9.70	3	n.f.	n.f.	n.f.	n.f.	n.f.	n.f.

Rny1p	n.f.	n.f.	n.f.	n.f.	18.58	6	n.f.	n.f.	6.84	3	9.82	2	18.65	5	n.f.	n.f.
Rpn11p	n.f.	n.f.	n.f.	n.f.	7.23	2	n.f.	n.f.	22.27	9	n.f.	n.f.	n.f.	n.f.	n.f.	n.f.
Scl1p	3.78	3	13.62	3	n.f.	n.f.	n.f.	n.f.	40.67	12	21.68	5	n.f.	n.f.	5.14	2
Scw11p	n.f.	n.f.	n.f.	n.f.	n.f.	n.f.	n.f.	n.f.	5.61	2	6.19	2	n.f.	n.f.	n.f.	n.f.
Sga1p	n.f.	n.f.	n.f.	n.f.	n.f.	n.f.	n.f.	n.f.	n.f.	n.f.	10.75	3	n.f.	n.f.	n.f.	n.f.
Ste23p	n.f.	n.f.	n.f.	n.f.	n.f.	n.f.	n.f.	n.f.	5.93	4	2.81	2	n.f.	n.f.	n.f.	n.f.
Ste24p	n.f.	n.f.	3.08	2	n.f.	n.f.	n.f.	n.f.	n.f.	n.f.	n.f.	n.f.	n.f.	n.f.	n.f.	n.f.
Ubp12p	n.f.	n.f.	n.f.	n.f.	n.f.	n.f.	n.f.	n.f.	29.97	11	8.68	3	n.f.	n.f.	n.f.	n.f.
Ubp14p	n.f.	n.f.	n.f.	n.f.	n.f.	n.f.	n.f.	n.f.	8.45	4	n.f.	n.f.	n.f.	n.f.	n.f.	n.f.
Ubp15p	n.f.	n.f.	n.f.	n.f.	n.f.	n.f.	n.f.	n.f.	52.17	19	6.92	4	n.f.	n.f.	n.f.	n.f.
Ubp1p	n.f.	n.f.	n.f.	n.f.	n.f.	n.f.	n.f.	n.f.	n.f.	n.f.	4.42	2	n.f.	n.f.	n.f.	n.f.
Ubp2p	n.f.	n.f.	n.f.	n.f.	n.f.	n.f.	n.f.	n.f.	16.11	8	2.21	4	n.f.	n.f.	n.f.	n.f.
Ubp3p	n.f.	n.f.	n.f.	n.f.	n.f.	n.f.	n.f.	n.f.	14.46	5	n.f.	n.f.	n.f.	n.f.	n.f.	n.f.
Ubp6p	n.f.	n.f.	4.67	2	n.f.	n.f.	n.f.	n.f.	26.57	10	n.f.	n.f.	n.f.	n.f.	n.f.	n.f.
Ubp7p	n.f.	n.f.	n.f.	n.f.	n.f.	n.f.	n.f.	n.f.	2.72	2	n.f.	n.f.	n.f.	n.f.	n.f.	n.f.
Ulp2p	n.f.	n.f.	n.f.	n.f.	n.f.	n.f.	n.f.	n.f.	3.42	2	n.f.	n.f.	n.f.	n.f.	n.f.	n.f.
Vma1p	72.83	36	602.63	51	269.1	49	n.f.	n.f.	309.81	70	276.22	58	145.55	35	14.78	4
Xrn1p	n.f.	n.f.	n.f.	n.f.	n.f.	n.f.	n.f.	n.f.	2.48	2	n.f.	n.f.	n.f.	n.f.	n.f.	n.f.
Ybl055cp	n.f.	n.f.	2.41	2	n.f.	n.f.	n.f.	n.f.	n.f.	n.f.	n.f.	n.f.	n.f.	n.f.	n.f.	n.f.
Ybr139wp	n.f.	n.f.	10.93	2	n.f.	n.f.	n.f.	n.f.	21.02	6	6.37	2	n.f.	n.f.	n.f.	n.f.
Yil108wp	n.f.	n.f.	n.f.	n.f.	n.f.	n.f.	n.f.	n.f.	6.14	3	n.f.	n.f.	n.f.	n.f.	n.f.	n.f.
Yol057wp	n.f.	n.f.	n.f.	n.f.	n.f.	n.f.	n.f.	n.f.	21.44	10	n.f.	n.f.	n.f.	n.f.	n.f.	n.f.
Yps1p	n.f.	n.f.	n.f.	n.f.	n.f.	n.f.	n.f.	n.f.	5.42	2	n.f.	n.f.	n.f.	n.f.	n.f.	n.f.
Yps3p	n.f.	n.f.	n.f.	n.f.	n.f.	n.f.	n.f.	n.f.	2.87	2	n.f.	n.f.	n.f.	n.f.	n.f.	n.f.
Yps7p	n.f.	n.f.	n.f.	n.f.	n.f.	n.f.	n.f.	n.f.	7.37	4	n.f.	n.f.	n.f.	n.f.	n.f.	n.f.
Yta12p	n.f.	n.f.	n.f.	n.f.	n.f.	n.f.	n.f.	n.f.	n.f.	n.f.	2.47	2	n.f.	n.f.	n.f.	n.f.
Yuh1p	3.85	3	n.f.	n.f.	n.f.	n.f.	n.f.	n.f.	24.14	6	18.43	4	n.f.	n.f.	n.f.	n.f.
