# Supplementary material for: First Proteomic Approach to Identify Cell Death Biomarkers in Wine Yeasts during Sparkling Wine Production
Source: Microorganisms. 2019 Nov 8;7(11):542. doi: 10.3390/microorganisms7110542 (PMC6920952; doi:10.3390/microorganisms7110542)
Supplement: Supplementary file 1 [file microorganisms-07-00542-s001.zip › Table S2.pdf]

Table S2. List of the most relevant autolysis-related proteins identified in *S. cerevisiae* P29 and G1 under each study condition (PC: pressure condition, NPC: non-pressure condition) and sampling time (T1: middle of the second fermentation, T2: one month after it). Proteins showing high content are highlighted in bold. a-g Different superscript letters indicate significant differences of protein content in each condition at 0.05 level according to Fisher's least significant difference procedure. Proteins showing 4-8 homogeneous groups (HG) are marked with an asterisk *. n.f.; not found. ns.; not significant.

**Condition**		**PCT1**				**PCT2**				**NPCT1**				**NPCT2**			
**Strain**		***P29***		***G1***		***P29***		***G1***		***P29***		***G1***		***P29***		***G1***	
**Protein**	**Molecular function**	[h]***mol%***	[i]***SD***	***mol%***	***SD***	***mol%***	***SD***	***mol%***	***SD***	***mol%***	***SD***	***mol%***	***SD***	***mol%***	***SD***	***mol%***	***SD***
Aap1p	Alanine/arginine aminopeptidase	0.011a	0.005	0.031b	0.006	n.f.a		n.f.a		0.07c	0.04	0.05bc	0.01	n.f.a		0.0391b	0.0007
Ai4p	Intron-encoded DNA endonuclease	n.f.a		n.f.a		n.f.a		n.f.a		0.014b	0.007	0.015b	0.005	n.f.a		n.f.a	
Ai5_Alphap	Intron-encoded DNA endonuclease	n.f.ns		n.f.ns		n.f.ns		n.f.ns		n.f.ns		0.011ns	0.008	n.f.ns		n.f.ns	
Ams1p	Alpha-mannosidase	n.f.a		n.f.a		0.022b	0.001	n.f.a		0.05c	0.02	n.f.a		n.f.a		n.f.a	
**Ape1p***	Vacuolar aminopeptidase 1	0.107c	0.004	0.16d	0.01	0.15d	0.02	n.f.a		0.098c	0.003	0.05b	0.03	0.183de	0.005	0.22e	0.04
**Ape2p***	Aminopeptidase 2, mitochondrial	0.009a	0.006	0.07c	0.02	n.f.ab		n.f.ab		0.06c	0.03	0.02b	0.01	n.f.ab		0.11d	0.01
**Ape3p***	Aminopeptidase Y	0.081b	0.001	0.24d	0.03	0.22d	0.05	n.f.a		0.083b	0.001	0.14c	0.04	0.30e	0.03	0.20d	0.01
Ape4p	Aspartyl aminopeptidase 4	n.f.a		n.f.a		n.f.a		n.f.a		0.019b	0.007	0.014b	0.007	n.f.a		n.f.a	
Arx1p	Probable metalloprotease	n.f.a		n.f.a		n.f.a		n.f.a		0.05b	0.02	n.f.a		n.f.a		n.f.a	
Atg4p	Cysteine protease	n.f.a		n.f.a		n.f.a		n.f.a		0.04c	0.01	0.02b	0.01	n.f.a		n.f.a	
Axl1p	Putative protease	n.f.ns		n.f.ns		n.f.ns		n.f.ns		0.006ns	0.001	0.004ns	0.002	n.f.ns		n.f.ns	
**Bgl2p***	Glucan 1,3-beta-glucosidase	0.36b	0.04	0.239a	0.005	1.0c	0.1	1.31d	0.02	0.26a	0.05	0.242a	0.003	1.3d	0.1	1.28d	0.01
Cps1p	Carboxypeptidase S	0.02a	0.01	0.08b	0.01	n.f.a		n.f.a		0.09b	0.05	0.06b	0.01	n.f.a		n.f.a	
Cym1p	Mitochondrial presequence protease	n.f.ns		n.f.ns		n.f.ns		n.f.ns		0.006ns	0.004	0.004ns	0.003	n.f.ns		n.f.ns	
Dap2p	Dipeptidyl aminopeptidase B	n.f.ns		n.f.ns		n.f.ns		n.f.ns		0.012ns	0.009	0.006ns	0.004	n.f.ns		n.f.ns	
Dcw1p	Mannan endo-1,6-alpha-mannosidase	n.f.a		n.f.a		n.f.a		n.f.a		0.04b	0.02	0.03b	0.01	n.f.a		n.f.a	

Dfg5p	Mannan endo-1,6-alpha-mannosidase	n.f.a		n.f.a		n.f.a		n.f.a		0.03c	0.01	0.02b	0.01	n.f.a		n.f.a	
Dna2p	ATP-dependent helicase/nuclease	0.007b	0.002	n.f.a		n.f.a		n.f.a		n.f.a		n.f.a		n.f.a		n.f.a	
Dse4p	Endo-1,3(4)-beta-glucanase 1	0.05a	0.02	n.f.a		n.f.a		n.f.a		0.16b	0.07	0.05a	0.04	n.f.a		0.11b	0.01
Dss1p	Exoribonuclease II, mitochondrial	n.f.ns		n.f.ns		n.f.ns		n.f.ns		0.004ns	0.003	0.004ns	0.003	n.f.ns		n.f.ns	
**Dug1p***	Cys-Gly metallodipeptidase	0.225cd	0.007	0.25d	0.05	0.04a	0.02	n.f.a		0.14b	0.03	0.17bc	0.02	0.20cd	0.02	0.8e	0.1
Dug2p	Probable di- and tripeptidase	n.f.a		n.f.a		n.f.a		n.f.a		0.02b	0.01	n.f.a		n.f.a		n.f.a	
Ecm14p	Putative metallocarboxypeptidase	n.f.a		n.f.a		n.f.a		n.f.a		0.013b	0.004	n.f.a		n.f.a		n.f.a	
Ecm38p	Gamma-glutamyltransferase	n.f.ns		n.f.ns		n.f.ns		n.f.ns		0.007ns	0.005	n.f.ns		n.f.ns		n.f.ns	
Esp1p	Separin	n.f.ns		0.007ns	0.004	n.f.ns		n.f.ns		0.005ns	0.002	n.f.ns		n.f.ns		n.f.ns	
**Exg1p***	Glucan 1,3-beta-glucosidase I/II	0.32c	0.02	0.25b	0.02	0.18a	0.05	1.0f	0.1	0.258bc	0.004	0.20ab	0.02	0.42d	0.06	0.80e	0.07
Exg2p	Glucan 1,3-beta-glucosidase 2	n.f.a		0.03c	0.01	n.f.a		n.f.a		0.015b	0.006	0.010b	0.004	n.f.a		n.f.a	
Fra1p	Putative Xaa-Pro aminopeptidase	0.013c	0.001	n.f.a		n.f.a		n.f.a		0.009b	0.003	n.f.a		n.f.a		n.f.a	
Lap2p	Leukotriene A-4 hydrolase homolog	n.f.a		0.0157b	0.0002	n.f.a		n.f.a		0.03c	0.02	0.0167b	0.0004	n.f.a		n.f.a	
Lap3p	Cysteine proteinase 1, mitochondrial	n.f.a		0.05b	0.01	n.f.a		n.f.a		0.06b	0.03	0.019a	0.007	n.f.a		n.f.a	
**Map1p**	Methionine aminopeptidase 1	0.22b	0.08	n.f.a		n.f.a		n.f.a		0.5c	0.1	0.17b	0.09	n.f.a		n.f.a	
Mas1p	Mitochondrial-processing peptidase subunit beta	n.f.a		0.06c	0.01	n.f.a		n.f.a		0.015a	0.007	0.03b	0.02	n.f.a		n.f.a	
**Mas2p**	Mitochondrial-processing peptidase subunit alpha	n.f.a		0.06b	0.01	n.f.a		n.f.a		0.02a	0.01	0.017a	0.005	n.f.a		0.13c	0.06
**Mca1p***	Metacaspase-1	n.f.a		n.f.a		0.11c	0.01	n.f.a		0.05b	0.02	0.05b	0.02	0.137d	0.008	n.f.a	
Nma111p	Pro-apoptotic serine protease	n.f.ns		0.02ns	0.01	n.f.ns		n.f.ns		0.011ns	0.006	n.f.ns		n.f.ns		n.f.ns	
Otu1p	Ubiquitin thioesterase	n.f.a		n.f.a		n.f.a		n.f.a		0.04b	0.02	0.018a	0.007	n.f.a		n.f.a	
**Pep4p***	Saccharopepsin	0.46bc	0.02	0.42b	0.04	0.43b	0.01	1.6e	0.1	0.2a	0.1	0.31a	0.05	0.6c	0.1	1.1d	0.1
Pim1p	Lon protease homolog, mitochondrial	n.f.a		n.f.a		n.f.a		n.f.a		0.018c	0.004	0.006b	0.004	n.f.a		n.f.a	
Prb1p*	Cerevisin	0.07cd	0.02	0.080de	0.001	0.098f	0.002	0.086ef	0.003	0.050ab	0.001	0.047a	0.002	0.12g	0.01	0.061bc	0.001
**Prc1p***	Carboxypeptidase Y	0.11b	0.02	0.19c	0.04	n.f.a		0.9f	0.1	0.145b	0.005	0.30d	0.04	n.f.a		0.71e	0.03
Prd1p	Saccharolysin	0.03b	0.02	0.04b	0.01	n.f.a		n.f.a		0.08c	0.01	0.018a	0.005	n.f.a		n.f.a	
Pre10p	Probable proteasome subunit alpha type-7	n.f.ns		0.11ns	0.08	n.f.ns		n.f.ns		0.04ns	0.03	n.f.ns		n.f.ns		n.f.ns	
Pre1p	Proteasome subunit beta type-4	n.f.ns		n.f.ns		n.f.ns		n.f.ns		0.07ns	0.04	0.04ns	0.02	n.f.ns		n.f.ns	

Pre2p	Proteasome subunit beta type-5	n.f.a		n.f.a		n.f.a		n.f.a		0.06b	0.03	n.f.a		n.f.a		n.f.a	
**Pre3p**	Proteasome subunit beta type-1	0.16c	0.04	0.16c	0.01	0.092b	0.004	n.f.a		0.103b	0.006	0.09b	0.03	n.f.a		0.16c	0.01
Pre4p	Proteasome subunit beta type-7	n.f.a		0.06b	0.03	n.f.a		n.f.a		0.06b	0.04	0.024a	0.001	n.f.a		n.f.a	
Pre5p*	Proteasome subunit alpha type-6	0.08c	0.03	0.07c	0.02	n.f.a		n.f.a		0.14d	0.02	0.037b	0.006	n.f.a		n.f.a	
**Pre6p***	Proteasome subunit alpha type-4	n.f.a		0.06c	0.01	0.07c	0.02	n.f.a		0.101d	0.006	0.042b	0.002	n.f.a		n.f.a	
**Pre7p**	Proteasome subunit beta type-6	n.f.a		n.f.a		0.2c	0.1	n.f.a		0.06b	0.01	0.03ab	0.01	n.f.a		n.f.a	
Pre8p*	Proteasome subunit alpha type-2	0.08b	0.02	0.08b	0.02	0.16d	0.03	n.f.a		0.119c	0.003	0.11c	0.01	0.120c	0.006	n.f.a	
**Pre9p***	Proteasome subunit alpha type-3	0.145de	0.001	0.16e	0.03	0.078c	0.004	n.f.a		0.133de	0.002	0.125d	0.002	0.05b	0.02	n.f.a	
Pup1p	Proteasome subunit beta type-2	n.f.ns		n.f.ns		n.f.ns		n.f.ns		0.05ns	0.03	0.03ns	0.02	n.f.ns		n.f.ns	
**Pup2p**	Proteasome subunit alpha type-5	0.30c	0.04	0.171b	0.003	n.f.a		n.f.a		0.19b	0.06	0.180b	0.005	0.08a	0.05	n.f.a	
**Pup3p***	Proteasome subunit beta type-3	0.102d	0.007	0.072b	0.004	n.f.a		n.f.a		0.14e	0.02	0.083c	0.002	n.f.a		n.f.a	
Rad2p	DNA repair protein	n.f.ns		n.f.ns		n.f.ns		n.f.ns		0.004ns	0.002	n.f.ns		n.f.ns		n.f.ns	
Rai1p	Decapping nuclease	n.f.ns		n.f.ns		n.f.ns		n.f.ns		0.009ns	0.006	n.f.ns		n.f.ns		n.f.ns	
Rex2p	Oligoribonuclease, mitochondrial	n.f.a		n.f.a		n.f.a		n.f.a		0.03b	0.01	n.f.a		n.f.a		n.f.a	
Rny1p*	Ribonuclease T2-like	n.f.a		n.f.a		0.100e	0.003	n.f.a		0.016c	0.004	0.011b	0.004	0.08d	0.01	n.f.a	
**Rpn11p**	Ubiquitin carboxyl-terminal hydrolase	n.f.a		n.f.a		0.04b	0.01	n.f.a		0.07c	0.01	n.f.a		n.f.a		n.f.a	
Scl1p	Proteasome subunit alpha type-1	0.05b	0.02	0.06b	0.01	n.f.a		n.f.a		0.11c	0.03	0.041ab	0.001	n.f.a		0.12c	0.05
Scw11p	Probable family 17 glucosidase	n.f.ns		n.f.ns		n.f.ns		n.f.ns		0.05ns	0.03	0.05ns	0.03	n.f.ns		n.f.ns	
Sga1p	Glucoamylase, intracellular sporulation-specific	n.f.a		n.f.a		n.f.a		n.f.a		n.f.a		0.03b	0.01	n.f.a		n.f.a	
Ste23p	A-factor-processing enzyme	n.f.ns		n.f.ns		n.f.ns		n.f.ns		0.008ns	0.005	0.004ns	0.003	n.f.ns		n.f.ns	
**Ste24p**	CAAX prenyl protease 1	n.f.ns		0.03ns	0.02	n.f.ns		n.f.ns		n.f.ns		n.f.ns		n.f.ns		n.f.ns	
Ubp12p	Ubiquitin carboxyl-terminal hydrolase 12	n.f.a		n.f.a		n.f.a		n.f.a		0.03b	0.01	0.007a	0.005	n.f.a		n.f.a	
Ubp14p	Ubiquitin carboxyl-terminal hydrolase 14	n.f.ns		n.f.ns		n.f.ns		n.f.ns		0.014ns	0.007	n.f.ns		n.f.ns		n.f.ns	
Ubp15p	Ubiquitin carboxyl-terminal hydrolase 15	n.f.a		n.f.a		n.f.a		n.f.a		0.04b	0.02	0.008a	0.004	n.f.a		n.f.a	
Ubp1p	Ubiquitin carboxyl-terminal hydrolase 1	n.f.ns		n.f.ns		n.f.ns		n.f.ns		n.f.ns		0.006ns	0.004	n.f.ns		n.f.ns	
Ubp2p	Ubiquitin carboxyl-terminal hydrolase 2	n.f.ns		n.f.ns		n.f.ns		n.f.ns		0.013ns	0.004	0.007ns	0.005	n.f.ns		n.f.ns	
Ubp3p	Ubiquitin carboxyl-terminal hydrolase 3	n.f.a		n.f.a		n.f.a		n.f.a		0.012b	0.006	n.f.a		n.f.a		n.f.a	

Ubp6p	Ubiquitin carboxyl-terminal hydrolase 6	n.f.a		0.018b	0.007	n.f.a		n.f.a		0.04c	0.02	n.f.a		n.f.a		n.f.a	
Ubp7p	Ubiquitin carboxyl-terminal hydrolase 7	n.f.a		n.f.a		n.f.a		n.f.a		0.004b	0.002	n.f.a		n.f.a		n.f.a	
Ulp2p	Ubiquitin-like-specific protease 2	n.f.ns		n.f.ns		n.f.ns		n.f.ns		0.004ns	0.003	n.f.ns		n.f.ns		n.f.ns	
**Vma1p***	V-type proton ATPase catalytic subunit A	0.170bc	0.007	0.3de	0.1	0.33e	0.02	n.f.a		0.169bc	0.004	0.14b	0.04	0.21cd	0.01	0.06a	0.02
Xrn1p	5'-3' exoribonuclease 1	n.f.a		n.f.a		n.f.a		n.f.a		0.003b	0.001	n.f.a		n.f.a		n.f.a	
Ybl055cp	Deoxyribonuclease Tat-D	n.f.a		0.02b	0.01	n.f.a		n.f.a		n.f.a		n.f.a		n.f.a		n.f.a	
Ybr139wp	Putative serine carboxypeptidase	n.f.a		0.04c	0.01	n.f.a		n.f.a		0.05c	0.02	0.015b	0.004	n.f.a		n.f.a	
Yil108wp	Putative zinc metalloproteinase	n.f.a		n.f.a		n.f.a		n.f.a		0.010b	0.005	n.f.a		n.f.a		n.f.a	
Yol057wp	Probable dipeptidyl peptidase 3	n.f.ns		n.f.ns		n.f.ns		n.f.ns		0.03ns	0.02	n.f.ns		n.f.ns		n.f.ns	
Yps1p	Aspartic proteinase 3	n.f.a		n.f.a		n.f.a		n.f.a		0.03b	0.01	n.f.a		n.f.a		n.f.a	
Yps3p	Aspartic proteinase yapsin-3	n.f.ns		n.f.ns		n.f.ns		n.f.ns		0.011ns	0.008	n.f.ns		n.f.ns		n.f.ns	
Yps7p	Aspartic proteinase yapsin-7	n.f.ns		n.f.ns		n.f.ns		n.f.ns		0.03ns	0.02	n.f.ns		n.f.ns		n.f.ns	
Yta12p	Mitochondrial respiratory complexes assembly	n.f.ns		n.f.ns		n.f.ns		n.f.ns		n.f.ns		0.004ns	0.003	n.f.ns		n.f.ns	
Yuh1p	Ubiquitin carboxyl-terminal hydrolase	0.109	0.002	n.f.a		n.f.a		n.f.a		0.1061	0.0003	0.07	0.04	n.f.a		n.f.a	

[h]Average values of protein content (mol %) and [i]standard deviation (SD).
